# Tranditional Roux-en-Y *vs* Uncut Roux-en-Y in Laparoscopic Distal Gastrectomy: a Randomized Controlled Study

**DOI:** 10.1007/s11605-023-05644-6

**Published:** 2023-03-14

**Authors:** Huahao Xie, Feng Wu, Chenshen Huang, Quanning Chen, Zhizhan Ni, Song Wang, Bujun Ge, Liming Liu, Qi Huang

**Affiliations:** 1 Department of General Surgery, Tongji Hospital, School of Medicine, Tongji University, Shanghai, China; 2 Department of General Surgery, Hwa Mei Hospital, University of Chinese Academy of Sciences, Zhejiang, China; 3 Department of Gastrointestinal Surgery, Fujian Provincial Hospital, Shengli Clinical Medical College of Fujian Medical University, Fuzhou, China; 4 Department of General Surgery, Shanghai Jing’an Shibei Hospital, Shanghai, China

## Abstract

**Background:**

Traditional Roux-en-Y may cause Roux-en-Y stasis syndrome (RSS), and Uncut Roux-en-Y was proposed to solve this problem. However, because afferent loop recanalization may occur after surgery, its clinical application remains controversial. The purpose of this study was to compare the long-term outcomes of these two gastrointestinal reconstruction methods.

**Methods:**

A total of 108 patients who received laparoscopic-assisted distal gastrectomy (LADG) were enrolled; 57 were randomly divided into the Uncut Roux-en-Y (URY) group, and 51 were divided into the Roux-en-Y (RY) group. Patients were followed up for 1 year to evaluate variables, including the following: (1) Assessments for RSS; (2) Preoperative and postoperative Gastrointestinal Symptom Rating Scale (GSRS) scores; (3) Postoperative gastroscopy to assess the occurrence of reflux esophagitis (Los Angeles classification), residual gastritis and bile reflux 1 year after surgery; and (4) Upper gastrointestinal radiography to evaluate whether recanalization occurred in patients in the URY group after surgery.

**Results:**

At 1 year after surgery, a total of 42 patients (73.7%) developed afferent loop recanalization. The incidence of RSS was not different between the two groups (OR, 1.301 [95% CI, 0.482 to 3.509]; *P* = 0.603*P* = 0.603). The GSRS score was higher in the URY group (*P* < 0.001). Postoperative gastroscopy showed that the incidence of bile reflux (*P* < 0.001) and the grade of residual gastritis (*P* < 0.001) were significantly higher in the URY group, but the grade of reflux esophagitis was not significantly different (*P* = 0.447, [95% CI, 0.437 to 0.457]*P* = 0.397).

**Conclusions:**

Compared with traditional Roux-en-Y anastomosis, due to the high recanalization rate, the URY group developed more severe gastrointestinal symptoms, the incidence of bile reflux and the grade of residual gastritis increased and the incidence of postoperative RSS was not reduced.

## Introduction

The most common surgical approach for gastric antrum malignancies is radical gastrectomy. The standard surgical approach is distal gastrectomy (DG) combined with D2 lymphadenectomy.^1,2^ As technology advances, laparoscopy-assisted distal gastrectomy (LADG) has gained popularity for general surgeons because of better postoperative outcomes.^3^

Gastrointestinal anastomosis is essential for gastric cancer surgery. Compared with Billroth I and Billroth II, several studies have reported that Roux-en-Y can effectively reduce the incidence of residual gastritis and esophagitis,^4–8^ surgical complications,^9,10^ and the recurrence rate of gastric cancer.^11^ However, traditional Roux-en-Y anastomosis still has its own disadvantages; approximately 30% of patients have postoperative symptoms associated with RSS,^12,13^ including epigastric distention, nausea and vomiting.^14^ The main reasons related to the continuity of the jejunum were blockages and changes in small intestine electrical conduction.^15,16^ To further improve the surgical method, some scholars developed Uncut Roux-en-Y anastomosis; this technique blocks the afferent loop, which is approximately 3 cm from the gastrojejunostomy.^17,18^ However, according to previous studies, the afferent limb recanalization rate ranges from 2.9% to 35.7% after Uncut Roux-en-Y anastomosis.^19,20^

The purpose of this study was to investigate the recanalization rate of the afferent loop and compare these two anastomosis methods in terms of long-term results, including RSS, reflux esophagitis, bile reflux and residual gastritis.

## Methods

### Ethics

This was a prospective, two-center, two-arm randomized controlled trial (RCT). Ethics approval was obtained from Shanghai Tongji Hospital (number: 2018-LCYJ-005), and approval was given from another center as needed. All patients provided written informed consent. The registration number is ChiCTR-1800015228.

### Patients

From June 2018 to December 2021, 140 adult patients who were scheduled to undergo laparoscopy-assisted distal gastrectomy (LADG) with D2 lymphadenectomy were recruited from Shanghai Tongji Hospital and Ningbo Hwa Mei Hospital. Twenty-three patients were excluded based on the exclusion criteria. The 117 included patients were randomly assigned (1:1) into two groups. Fifty-nine patients underwent Uncut Roux-en-Y surgery in the study group (URY group), and 58 patients underwent traditional Roux-en-Y surgery in the control group (RY group). During the postoperative follow-up, two patients died after discharge in the URY group, and 7 patients were lost to follow-up in the RY group. Ultimately, 57 patients were included in the URY group, and 51 patients were included in the RY group. All surgeons had surgical experience with at least 100 LADG surgeries.

**Fig. 1:**
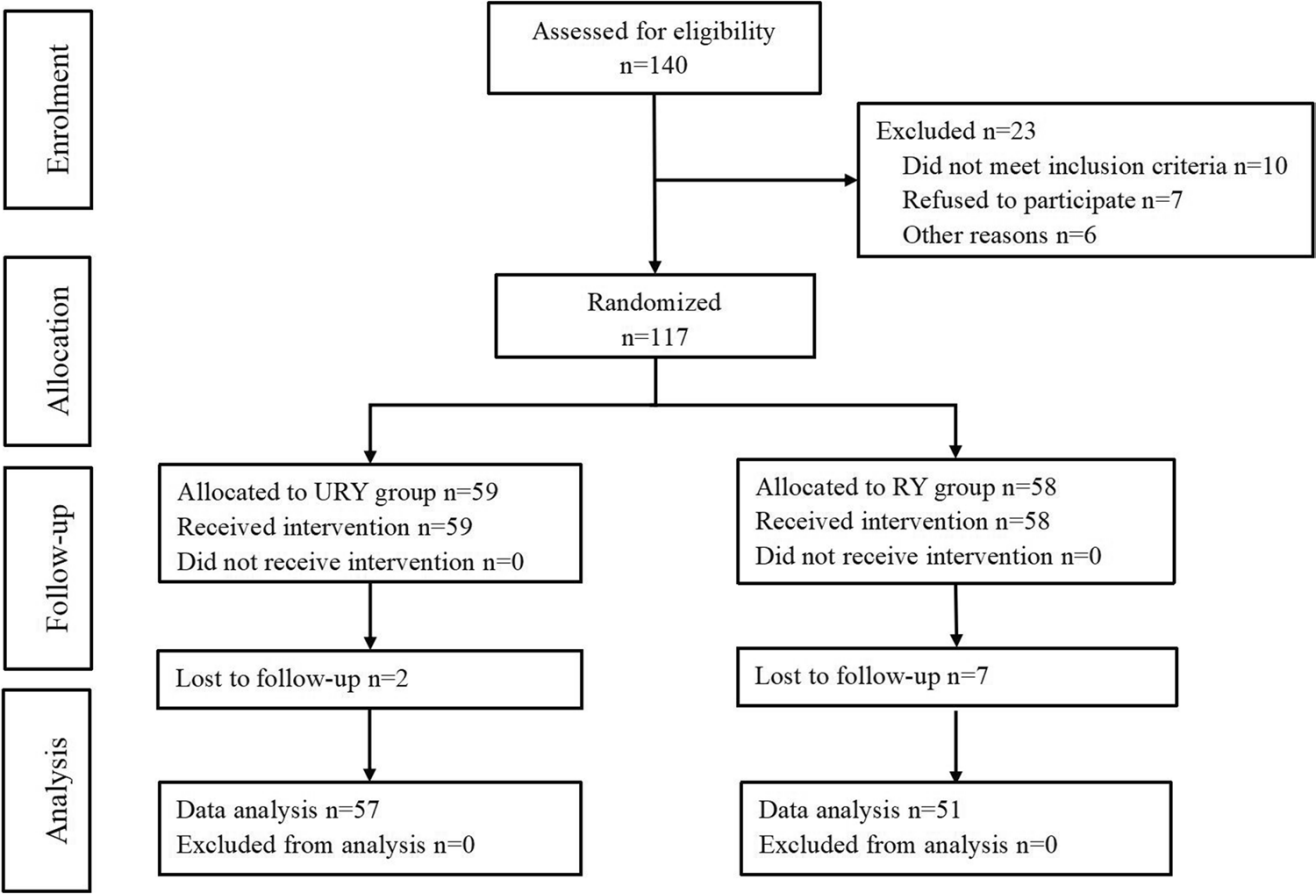
Consort diagram for the study

The inclusion criteria were as follows: (1) patients aged ≥ 18 years; (2) patients with endoscopic biopsy and pathology reports confirming primary gastric cancer; (3) patients with stage I-III clinical tumors; (4) patients with ECOG scores of 0–1; and (5) patients with American Society of Anesthesiologists (ASA) grade I—III tumors. The exclusion criteria were as follows: (1) unstable angina, myocardial infarction, or cerebrovascular events within the past 6 months; (2) various serious mental diseases; (3) emergency surgery or pyloric obstruction; (4) previous history of upper abdominal surgery; (5) neoadjuvant chemoradiotherapy before surgery; and (6) other malignant tumors. The scheme of the study process is shown in Fig. 1.

### Preoperative Management

All patients underwent gastroscopy, biopsy, and computed tomography (CT) to assess tumor characteristics. Other tests, including electrocardiogram, echocardiogram, and pulmonary function tests, were used to examine cardiopulmonary function. Patients fasted for 6 h and had no water for 2 h before surgery. General anesthesia was maintained by endotracheal intubation. Nasogastric and urinary catheters were placed before surgery.

### Surgical Approach

**Fig. 2:**
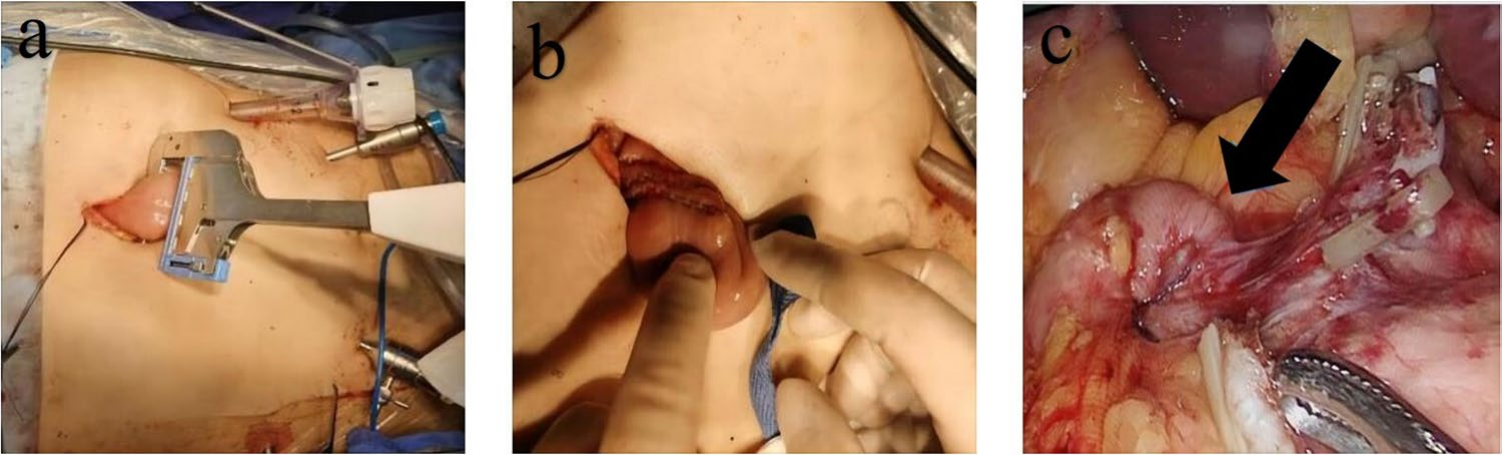
Uncut Roux-en-Y anastomosis procedure. (**a**) Closed afferent limb in vitro (6 rows of nails). (**b**) Afferent limb jejunum closed with a linear stapler. (**c**) Afferent limb (6 rows of nails, black arrow)

All enrolled patients underwent LADG combined with D2 lymphadenectomy. Roux-en-Y surgery was performed as described previously^21^. Uncut Roux-en-Y anastomosis was performed as follows: After closing the duodenal stump, side-to-side anastomosis of the stomach and jejunum was performed, and the distances with the ligament of Treitz were approximately 30 cm. Side-to-side anastomosis between the jejunum was performed approximately 35 cm from the gastrojejunostomy. Then, the afferent loop was blocked using 3 linear staplers with two rows of staples, and the length of the staplers used in the operation is 60 mm. (Fig. 2).

### Postoperative Management

According to enhanced recovery after surgery (ERAS) principles,^22^ postoperative analgesia, early catheter removal, and early initiation of walking were performed on postoperative Day 1 (POD 1). The nasogastric tube was removed on POD 2. Patients were encouraged to eat a semisolid diet based on tolerance on POD 3. The drain was removed within 3 to 4 days.

### Primary and Secondary Outcomes

The primary outcome was the incidence of RSS, which was assessed as follows: (1) Nausea, vomiting, and abdominal pain after surgery; (2) Refasting after return to a semifluid or normal diet; and (3) Readmission due to these reasons after surgery. Patients diagnosed with gastroparesis, intestinal paralysis, and anastomotic stenosis through clinical and image findings were not considered to have RSS.^23^ The secondary outcomes were preoperative and postoperative Gastrointestinal Symptom Rating Scale (GSRS) scores. Postoperative gastroscopy was used to assess the occurrence of the reflux esophagitis grade (Los Angeles classification), residual gastritis, and the degree of bile reflux at 1 year after surgery.

### Gastroscope Grading

The gastroscopic esophagitis grade criteria followed the Los Angeles Classification System:^24^ (1) Grade A refers to one (or more) esophageal mucosal breaks less than 5 mm; (2) Grade B refers to one (or more) esophageal mucosal breaks greater than 5 mm; (3) Grade C refers to one (or more) mucosal breaks that are continuous between the tops of two or more mucosal folds but involves less than 75% of the circumference; and (4) Grade D refers to one (or more) mucosal breaks that involve more than 75% of the circumference.

Residual gastritis was divided into four categories: 0, the same as the surrounding normal tissue; I, local hyperemia and edema of the residual gastric mucosa; II, mucosa with scattered or intermittent linear hyperemia and edema; and III, widespread residual gastric mucosa hyperemia and edema.

### Sample Size

The required sample size in each group was calculated using G*Power (University Kiel, Germany) software. There have been no exact incidences of RSS after RY or URY evaluated based on a large cohort. A minimum sample size of 44 patients per randomization arm was estimated to yield a statistical power of at least 0.8 with an alpha of 0.05 and a medium effect size (d = 0.3). Considering a loss to follow-up of up to 10%, at least 50 patients should be included in each group.

### Statistical Methods

IBM SPSS Statistics 26 statistical software was used for analysis. Student’s *t* test was used to compare continuous variables with a normal distribution. Categorical variables were compared by the chi-square test, and *P* values < 0.05 were considered statistically significant.

## Results

### Patient Characteristics and Perioperative Surgical Outcomes

After screening and excluding patients who dropped out and were lost to follow-up, 57 patients were included in the URY group and 51 patients were included in the RY group. Clinical data are shown in Table 1. There were no significant differences in sex (OR, 0.548 [95%CI, 0.240 to 1.255]; *P* = 0.153), age (66.16 ± 7.94 vs 66.34 ± 9.05; *P* = 0.380), tumor location (OR, 0.646 [95%CI, 0.275 to 1.521]; *P* = 0.316), preoperative GSRS symptoms (*P* = 0.114, [95%CI, 0.112 to 0.125]), gastroesophageal reflux symptoms (OR, 0.463 [95%CI, 0.209 to 1.026]; *P* = 0.056), or clinicopathological stages (*P* = 0.424, [95%CI, 0.415 to 0.434]).

**Table 1:** Clinical and pathological data of the patients

Variable	URY group	RY group	P*
	*n* = 57	*n* = 51	
Gender			0.153
Male	43(75.4%)	32(62.7%)	
Female	14(24.6%)	19(37.3%)	
Age	66.16 ± 7.94	66.34 ± 9.05	0.380†
Tumor location			0.316
Body of stomach	13(22.8%)	16(31.4%)	
Pylorus	44(77.2%)	35(68.6%)	
cTNM stage			0.424
I	20(35.1%)	16(31.4%)	
II	5(8.8%)	9(17.6%)	
III	32(56.1%)	26(51.0%)	
Preoperative GSRS			0.114
0	13(22.8%)	12(23.5%)	
1–3	27(47.4%)	15(29.4%)	
> 3	17(29.8%)	24(47.1%)	
Gastroesophageal reflux symptoms			0.056
positive	27(47.4%)	15(29.4%)	
negative	30(52.6%)	36(70.6%)	

*Pearson’s χ2 test, except †Student’s t test

The difference in the conversion rate from laparoscopic to open surgery and the hospital stay (17.49 ± 6.84 vs 16.84 ± 6.04; *P* = 0.605) were not statistically significant. The URY group had a longer duration of surgery (219.89 ± 40.27 vs 195.12 ± 39.70, *P* = 0.002) and a higher incidence of surgical complications (*P* = 0.024, [95%CI, 0.021 to 0.027]), mainly Clavien‒Dindo grades I-II, such as poor gastroparesis (3 cases), poor incision healing (2 cases), intestinal paralysis (1 case), pancreatic fistula (1 case), and lymphatic fistula (1 case) (Table 2).

**Table 2:** Intraoperative conditions and short-term postoperative outcomes

Variable	URY group	RY group	P*
	*n* = 57	*n* = 51	
Operation Time	219.89 ± 40.27	195.12 ± 39.70	0.002†
Transition to laparotomy			0.440
NO	48(84.2%)	40(78.4%)	
YES	9(15.8%)	11(21.6%)	
Postoperative complications (Clavien-Dindo grade)			0.024
0	41(71.9%)	46(90.2%)	
I	11(19.3%)	2(3.9%)	
II	5(8.8%)	3(5.9%)	
hospital stay	17.49 ± 6.84	16.84 ± 6.04	0.605†

*Pearson’s χ2 test, except †Student’s t test

### Recanalization Results

**Fig. 3:**
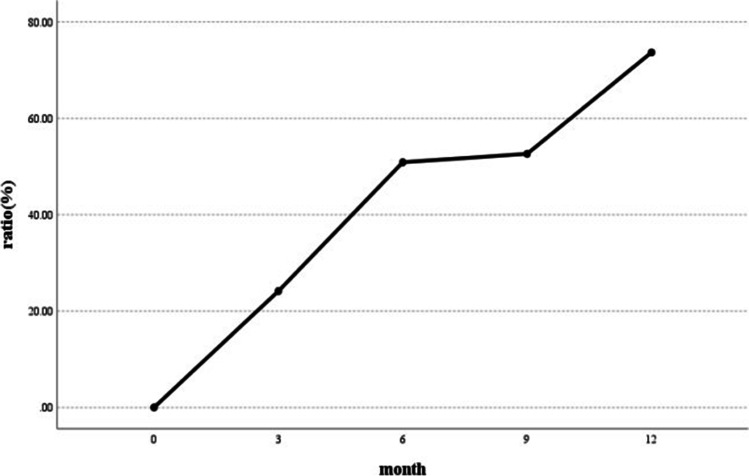
Changes in the recanalization rate after Uncut Roux-en-Y anastomosis

**Fig. 4:**
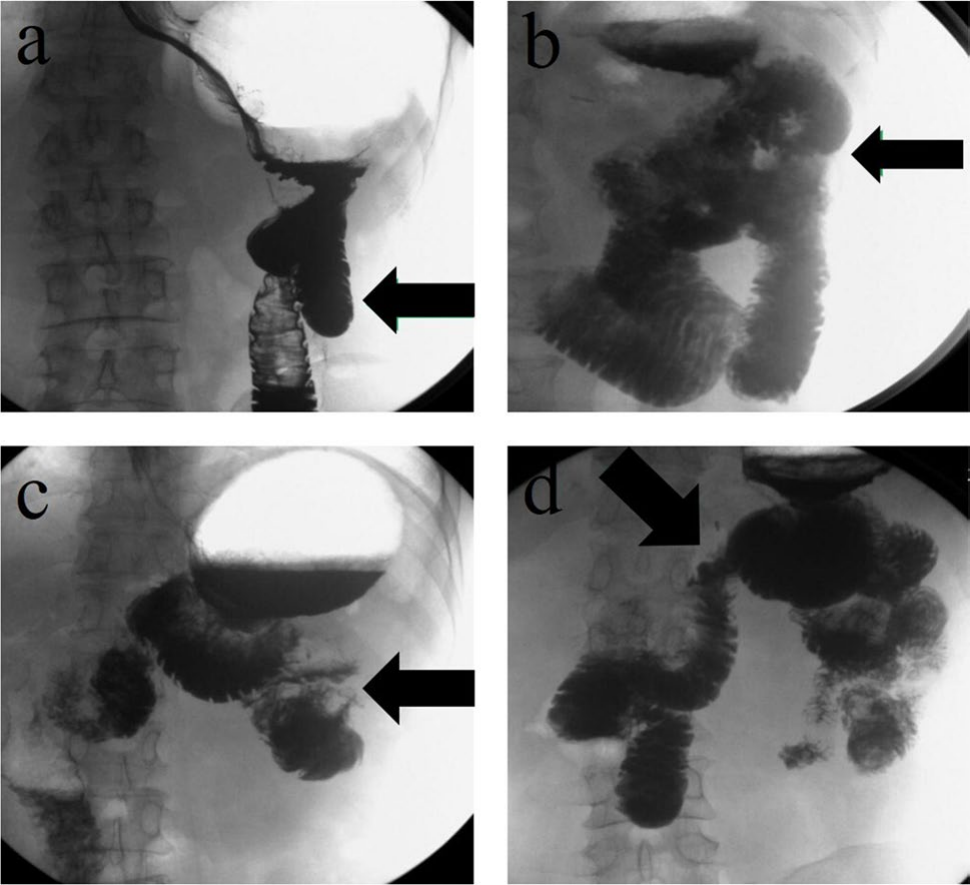
Upper gastrointestinal radiography after Uncut Roux-en-Y anastomosis. Upper gastrointestinal radiography showing the closure of the afferent limb (black arrows) 6 months after the operation (**a**) and recanalization (black arrow) 9 months after the operation (**b**). In another case, upper gastrointestinal radiography showing the closure of the afferent limb (black arrow) 9 months after the operation (**c**) and recanalization (black arrow) 12 months after the operation (**d**)

After a one-year follow-up, the cumulative number of recanalizations was 42 (73.7%) through upper gastrointestinal radiography (Fig. 3), and the recanalization of different periods is shown in Table 3. Upper gastrointestinal radiography after Uncut Roux-en-Y anastomosis was performed to evaluate whether patients were recanalized (Fig. 4).

**Table 3:** Recanalization in URY group

Postoperative (months)	Number of New Recanalization Cases	Cumulative Recanalization Cases	Cumulative Recanalization Ratio
3	14	14	24.6%
6	15	29	50.9%
9	1	30	52.6%
12	12	42	73.7%

### Follow-Up Results

The postoperative follow-up results of the two groups are shown in Table 4. Nine patients (15.8%) in the URY group and 10 patients (19.6%) in the RY group developed RSS, and the incidence of RSS did not differ (OR, 1.301 [95%CI, 0.482 to 3.509]; *P* = 0.603). The GSRS score of the URY group was substantially higher than that of the RY group (*P* < 0.001).

**Table 4:** Postoperative long-term follow-up results

Variable	URY group	RY group	P*
	*n* = 57	*n* = 51	
GSRS			< 0.001
0	13(22.8%)	37(72.5%)	
1–3	42(73.7%)	13(25.5%)	
> 3	2(3.5%)	1(2.0%)	
RSS			0.603
Positive	9(15.8%)	10(19.6%)	
Negative	48(84.2%)	41(80.4%)	
Reflux Esophagitis Grade (Gastroscopy)			0.447
0	39(68.4%)	30(58.8%)	
A	15(26.3%)	15(29.4%)	
B	3(5.3%)	6(11.8%)	
Residual Gastritis (Gastroscopy)			< 0.001
0	3(5.3%)	3(5.9%)	
I	37(64.9%)	48(94.1%)	
II	14(24.5%)	0	
III	3(5.3%)	0	
Bile Reflux (Gastroscopy)			< 0.001
Positive	38(66.7%)	3(5.9%)	
Negative	19(33.3%)	48(94.1%)	

*Pearson’s χ2 test, except †Student’s t test

**Fig. 5:**
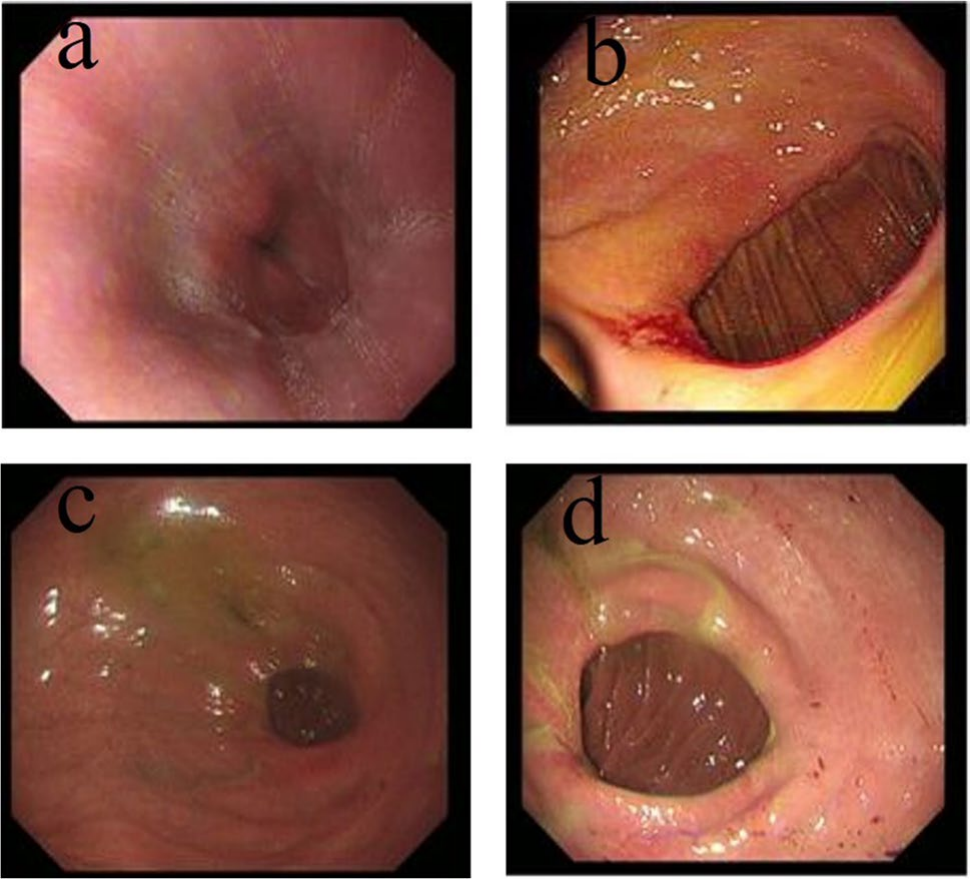
Gastroscopy in the URY group at 1 year after surgery. (**a**) Endoscopy showing esophageal mucosal hyperemia and edema. (**b**) Endoscopy showing residual gastric hyperemia and edema with ulcers and bile reflux seen around. (**c**) and (**d**) Both showing recanalization of the afferent limb, bile reflux, yellow‒green mucus, and anastomotic edema

Postoperative gastroscopy (Fig. 5) showed that there was no difference in reflux esophagitis grades (*P* = 0.447, [95%CI, 0.437 to 0.457]), but the grade of residual gastritis (*P* < 0.001) and the incidence of bile reflux (*P* < 0.001) in the URY group were significantly higher than those in the RY group.

### Subgroup Analysis

To explore the effect of recanalization on follow-up outcomes, patients in the URY group were assigned to the recanalization group (*n* = 42) and the nonrecanalization group (*n* = 15). The results are shown in Table 5. There was no difference in the grade of reflux esophagitis (*P* = 0.192, [95%CI, 0.184 to 0.200]), while the incidence of residual gastritis (*P* = 0.043, [95%CI, 0.046 to 0.054]) and the degree of bile reflux (OR, 4.800[95%CI, 1.370 to 16.812]; *P* = 0.011) were significantly higher in the recanalization group. No difference was observed in the incidence of RSS (OR, 0.304[95%CI, 0.035 to 2.659]; *P* = 0.474), but the GSRS score in the recanalization group was substantially higher than that in the RY group (*P* = 0.041, [95%CI, 0.037 to 0.044]).

**Table 5:** Comparison of subgroups based on recanalization situation in URY group

Variable	Recanalization group	Non-recanalization group	P*
	*n* = 42	*n* = 15	
GSRS			0.041
0	6(14.3%)	7(46.7%)	
1–3	34(81.0%)	8(53.3%)	
> 3	2(4.7%)	0	
RSS			0.474
Positive	8(19.0%)	1(6.7%)	
Negative	34(81.0%)	14(93.3%)	
Reflux Esophagitis Grade (Gastroscopy)			0.192
0	26(61.9%)	13(86.7%)	
A	13(31.0%)	2(13.3%)	
B	3(7.1%)	0	
Residual Gastritis (Gastroscopy)			0.043
0	1(2.4%)	2(13.3%)	
1	26(61.9%)	11(73.4%)	
2	12(28.6%)	2(13.3%)	
3	3(7.1%)	0	
Bile Reflux (Gastroscopy)			0.011
Positive	10(23.8%)	9(60.0%)	
Negative	32(76.2%)	6(40.0%)	

*Pearson’s χ2 test, except †Student’s t test

## Discussion

Uncut Roux-en-Y was invented to improve gastrointestinal reconstruction and reduce postoperative complications. However, in this trial, the incidence of RSS did not differ between the two groups. At the same time, the URY group showed a significantly higher incidence of residual gastritis and degree of bile reflux, and the GSRS score was obviously higher after surgery.

Uncut Roux-en-Y was invented by Van Stiegmann and Goff in 1988;^25^ this method does not cut off the jejunum, maintains the continuity of proximal jejunal anatomy and electrophysiological activity, avoids secondary pacemakers in the intestine, and helps relieve RSS symptoms.^26,27^ However, because afferent loop recanalization may occur after surgery, Uncut Roux-en-Y anastomosis has long been controversial. In our previous animal experiments with pigs, the incidence of recanalization reached 100% at 1 month after surgery.^28^ In this clinical study, the commonly used 6-row nails were used to close the afferent limb, and the recanalization rate was as high as 73.7%.

According to a previous study, closing the afferent loop jejunum may not result in permanent healing between the mucosa and the mucosa, which is the main reason for the recanalization of the bowel.^29–31^ This finding indicates that the linear stapler is not complete enough to close the afferent limb jejunum and needs to be further improved. Some scholars have proposed different methods of closing the afferent loop, and it has been reported that ligation with a 7-gauge silk thread can effectively reduce the recanalization rate. However, the strength of ligating the intestinal tube is not easy to grasp, and the ligation of the intestinal tube is too loose, which can lead to the occurrence of recanalization. There are also studies that improve this, with 6–8 layers of seromuscular layer in the upper and lower sections of the intestinal canal sutured at the closure, with the aim to further reduce the recanalization rate of the afferent limb.^20,32,33^

Generally, Uncut Roux-en-Y anastomosis involves Billroth II combined with Braun anastomosis, which prevents RSS after surgery.^34^ Many previous studies have analyzed the differences between Uncut Roux-en-Y and Billroth II and showed that Uncut Roux-en-Y can significantly decrease the incidence of residual gastritis and bile reflux.^18,35,36^ However, clinical trials of Uncut Roux-en-Y and Roux-en-Y are rarely reported. Our study found that the URY group did not show any advantages in preventing RSS. Moreover, the incidence of residual gastritis and the degree of bile reflux in the URY group were significantly higher than that in the RY group, which may be associated with the high recanalization rate, and our subgroup analysis also supports this conclusion.

Several limitations were identified in this study. First, the study design and sample size aimed to explore the advantages and disadvantages of these two reconstruction methods. When performing subgroup analysis, the results may not be sufficient for statistical analysis. Second, although many methods have been reported, blocking the afferent loop using a 6-row stapler is still the mainstream method.^37^ Therefore, this method was adopted in this study.

This study shows that the linear stapler cannot effectively block the recanalization of the afferent limb. Due to the high recanalization rate, the URY group developed more severe gastrointestinal symptoms, bile reflux, and had an increased residual gastritis incidence, and did not have a reduced incidence of postoperative RSS. Therefore, uncut Roux-en-Y reconstruction is not recommended before finding a way to reduce the recanalization rate.

## Data Availability

The raw data of this paper are available from the authors upon reasonable request.
